# *linc-20* and *linc-9* do not have compensatory fertility roles in *C. elegans*

**DOI:** 10.17912/micropub.biology.000524

**Published:** 2022-02-11

**Authors:** Yisrael Rappaport, Roni Falk, Hanna Achache, Yonatan B. Tzur

**Affiliations:** 1 Department of Genetics, Institute of Life Sciences, The Hebrew University of Jerusalem, Jerusalem 91904, Israel

## Abstract

Long intergenic non-coding RNAs (lincRNAs) are transcripts longer than 200 nucleotides which are transcribed from regions that do not overlap with protein coding sequences. Reproductive organs express high levels of lincRNAs, yet removal of many lincRNA genes with high and dynamic germline expression did not lead to fertility defects. It was previously suggested this stems from redundant roles of different lincRNA genes. We previously reported engineering *C. elegans *strains in which we deleted lincRNA genes with high and dynamic expression in the gonad. The individual mutations did not lead to major effects on fertility. Two of those lincRNA genes, *linc-9* and *linc-20,* are highly homologous, suggesting they could perform redundant roles. Here we report that in the double mutant *linc-9; linc-20 *the brood size and embryonic lethality do not significantly differ from wild-type worms. This could be explained by either lack of fertility roles, or redundancy with other lincRNA genes.

**Figure 1. Fertility analyses of linc-9; linc-20:**
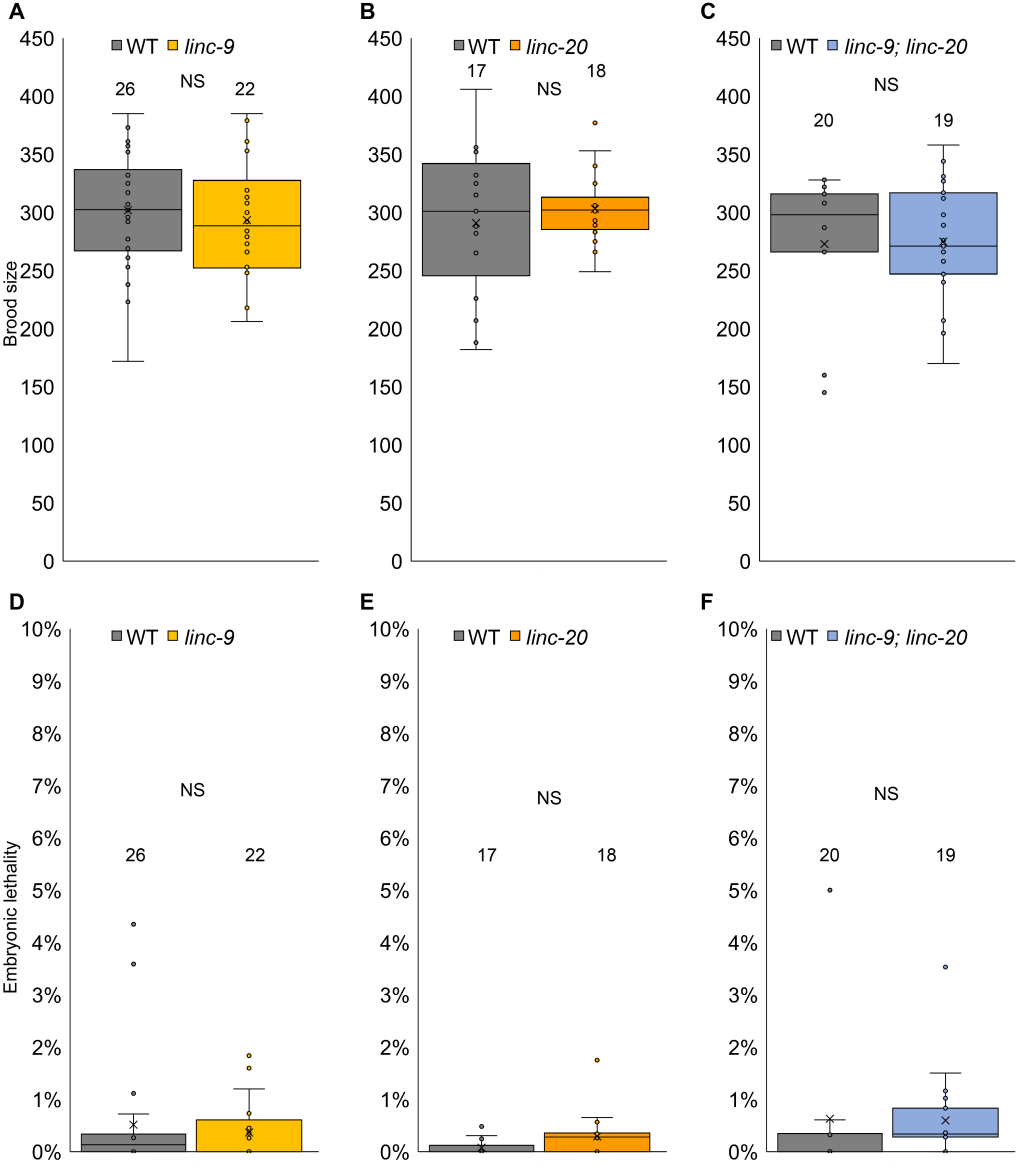
**A-C.** Box plots depict the average number (x), the median (horizontal line within the box) and the individual measurements (circles) of brood size per worm. **D-F.** Box plots depict the average number (x), the median (horizontal line within the box) and the individual measurements (circles) of embryonic lethality level per worm. Data for **A, B, D, E** was adapted from (Ishtayeh *et al.*, 2021). N values appear above each box plot. Mann-Whitney *p* value: NS – not significant.

## Description

Long non-coding RNAs (lncRNAs) are transcripts which are longer than 200 nucleotides which are not translated into proteins. These transcripts are usually transcribed by RNA Polymerase II, often capped, polyadenylated, and spliced (reviewed in (Blythe *et al.*, 2016; Deniz and Erman, 2017; Fatica and Bozzoni, 2014; Fico *et al.*, 2019; Kopp and Mendell, 2018; Marques and Ponting, 2014; Melissari and Grote, 2016; Shields *et al.*, 2019; Ulitsky and Bartel, 2013)). Long intergenic non-coding RNAs (lincRNAs) are a subclass of lncRNAs which are transcribed from genomic regions which do not code for proteins (Deniz and Erman, 2017). Many lincRNAs are transcribed during gametogenesis, and mammalian testis is the tissue with the highest number of dynamically expressed long non-coding RNAs (Soumillon *et al.*, 2013; Washietl *et al.*, 2014). In many metazoans reverse genetics analysis indicated that knockout or knockdown of lncRNAs which are dynamically expressed in reproductive organs did not lead to loss of fertility (Dai *et al.*, 2019; Ganesh *et al.*, 2020; Goudarzi *et al.*, 2019; Li *et al.*, 2020; Wei *et al.*, 2019 Ishtayeh, 2021 #8555; Zhang *et al.*, 2012; Zhou *et al.*, 2021; Zhu *et al.*, 2020). Several reports suggested this is due to redundancy in the action of two or more lncRNA genes (Goudarzi *et al.*, 2019; Ishtayeh *et al.*, 2021; Wichman *et al.*, 2017). To the best of our knowledge testing this hypothesis has not been published.

We previously reported the analysis of expression of lincRNA genes in the gonad of *C. elegans,* and the identification of six genes with high and dynamic expression (Ishtayeh *et al.*, 2021). We created strains with complete deletion of these genes using CRISPR. Compared to localized mutation within the gene, complete deletion ensures that no leftover transcript still maintains residual roles, and that transcriptional compensation is not triggered (El-Brolosy *et al.*, 2019; Rossi *et al.*, 2015; Serobyan *et al.*, 2020), thus reducing the possibility for misinterpretation of the roles of the mutated gene. We reported that we did not find any significant reduction in brood size in any of the six lincRNA deletion strains (Ishtayeh *et al.*, 2021). We also did not find any change in several meiotic processes we examined. In that report we noted that two of these lincRNAs, namely *linc-9* and *linc-20,* are highly homologous (over 90% identity) (Ishtayeh *et al.*, 2021). This raises the hypothesis that these lincRNAs genes have similar or redundant roles, and so when only one is deleted, no fertility effect can be detected.

To find if *linc-9* and *linc-20* have redundant roles in hermaphrodite’s fertility, we crossed the five times outcrossed individual deletion strains to create a *linc-9; linc-20* homozygous strain. To compare the fertility of this strain to wild-type worms, we singled L4 larvae on NGM plates, and transferred them to new plates every 24 hours for three days (see methods). We found no significant change in the brood size of the worms between wild type and *linc-9; linc-20* worms (Fig. 1, 273±14 and 275±12, n = 20,19 respectively, *p* value = 0.8 by the two tailed Mann-Whitney test). *linc-9; linc-20* brood was similar to those of the single mutants (compare Fig. 1 C to Fig. 1 A, B, and (Ishtayeh *et al.*, 2021)). This result suggests that just like the single mutations, the double mutation of *linc-9* and *linc-20* does not reduce *C. elegans* fertility under normal laboratory conditions.

Embryonic lethality (Emb phenotype) was used previously to identify meiotic aberrations (as well as other developmental defects). Neither *linc-9* nor *linc-20* led to increased embryonic lethality compared to wild-type worms (Ishtayeh *et al.*, 2021). To test if the combined *linc-9; linc-20* leads to increased embryonic lethality, we scored the percentage of unhatched embryos laid by wild-type vs. *linc-9; linc-20* worms. We found no significant change between the strains (Fig. 1B, 0.6%±0.3% and 0.6±%0.2%, n= 20,19 respectively, *p* value = 0.4 by the two tailed Mann-Whitney test). *linc-9; linc-20* embryonic lethality was similar to those of the single mutants (compare Fig. 1 F to Fig. 1 D, E, and (Ishtayeh *et al.*, 2021)). Therefore, the combined mutations do not lead to increased embryonic lethality.

Our results suggest that the double mutations in *linc-9* and *linc-20* do not result in a dramatic change in fertility, at least under our laboratory experimental conditions. These results still leave the open question of why these genes are expressed at high and dynamic pattern in the gonad (Ishtayeh *et al.*, 2021; Tzur *et al.*, 2018) if their complete removal does not lead to a change in fertility. Several answers can be suggested: It is possible that their roles are redundant with other genes with less sequence homology, or with unannotated homologous genes. Another possibility is that these transcripts’ germline expression has non-fertility related roles, or roles that manifest under different conditions as we have previously shown for *linc-4* (Ishtayeh *et al.*, 2021)*.* Compared to protein coding genes, lincRNAs genes are very poorly evolutionary conserved (Wei *et al.*, 2019), suggesting *linc-9* and *linc-20* may have been recently introduced into the genome, and not yet adopted biological roles or any biological role they used to have, has been inactivated. A recent review of the insights gained from reverse genetics experiments in lncRNAs suggested that in many cases removal of lncRNA genes lead to milder or no phenotype (Gao *et al.*, 2020). This strengthens the possibility that indeed these genes have no functional roles. Future research may provide more insights into the functional roles of *linc-9* and *linc-20* expression.

## Methods

***C. elegans* strains**

The N2 Bristol strain was utilized as the wild-type background. YBT60 was created by crossing YBT54 and YBT53 (Ishtayeh *et al.*, 2021). All strains were cultured under standard conditions at 20 °C (Brenner, 1974). Worms were grown on NGM plates with *Escherichia coli* OP50 (Brenner, 1974).

**Progeny quantification**

The brood size was determined by placing at least 17 individual L4 worms on seeded NGM plates, transferring each worm to a new plate every 24 h, and counting their embryos and hatched progeny over 3 days. Data for Fig. 1 A, B, D, E was adapted from (Ishtayeh *et al.*, 2021).

**Statistical Analysis**

Significance between mutant and wild type worms was tested by the Mann-Whitney test. Using the R package ‘wmwpow’ (Mollan *et al.*, 2020), we calculated the power for the two-sided Wilcoxon-Mann-Whitney test performed on our two specified distributions (n=20 for WT and n=19 for *linc-9: linc-20*) with an empirical p-value of 0.05. We found a power of 91%.

## Reagents

**Table:** 

Strain	Genotype	Shorten Name	Available from
Bristol N2	*Caenorhabditis elegans*	Wild type	CGC
YBT53	*linc-9(huj24)*	*linc-9*	Tzur lab
YBT54	*linc-20(huj21)*	*linc-20*	Tzur lab
YBT60	*linc-9(huj24); linc-20(huj21)*	*linc-9; linc-20*	Tzur lab

## References

[R1] Blythe AJ, Fox AH, Bond CS (2015). The ins and outs of lncRNA structure: How, why and what comes next?. Biochim Biophys Acta.

[R2] Brenner S (1974). The genetics of Caenorhabditis elegans.. Genetics.

[R3] Dai, Y., Lin, Y., Song, N., and Sun, F. (2019). LncRNA4667 is dispensable for spermatogenesis and fertility in mice. Reproductive and Developmental Medicine 3: 18-23.

[R4] Deniz E, Erman B (2016). Long noncoding RNA (lincRNA), a new paradigm in gene expression control.. Funct Integr Genomics.

[R5] El-Brolosy MA, Kontarakis Z, Rossi A, Kuenne C, Günther S, Fukuda N, Kikhi K, Boezio GLM, Takacs CM, Lai SL, Fukuda R, Gerri C, Giraldez AJ, Stainier DYR (2019). Genetic compensation triggered by mutant mRNA degradation.. Nature.

[R6] Fatica A, Bozzoni I (2013). Long non-coding RNAs: new players in cell differentiation and development.. Nat Rev Genet.

[R7] Fico A, Fiorenzano A, Pascale E, Patriarca EJ, Minchiotti G (2019). Long non-coding RNA in stem cell pluripotency and lineage commitment: functions and evolutionary conservation.. Cell Mol Life Sci.

[R8] Ganesh S, Horvat F, Drutovic D, Efenberkova M, Pinkas D, Jindrova A, Pasulka J, Iyyappan R, Malik R, Susor A, Vlahovicek K, Solc P, Svoboda P (2020). The most abundant maternal lncRNA Sirena1 acts post-transcriptionally and impacts mitochondrial distribution.. Nucleic Acids Res.

[R9] Gao F, Cai Y, Kapranov P, Xu D (2020). Reverse-genetics studies of lncRNAs-what we have learnt and paths forward.. Genome Biol.

[R10] Goudarzi M, Berg K, Pieper LM, Schier AF (2019). Individual long non-coding RNAs have no overt functions in zebrafish embryogenesis, viability and fertility.. Elife.

[R11] Ishtayeh H, Achache H, Kroizer E, Rappaport Y, Itskovits E, Gingold H, Best C, Rechavi O, Tzur YB (2020). Systematic analysis of long intergenic non-coding RNAs in *C. elegans* germline uncovers roles in somatic growth.. RNA Biol.

[R12] Kopp F, Mendell JT (2018). Functional Classification and Experimental Dissection of Long Noncoding RNAs.. Cell.

[R13] Li C, Shen C, Shang X, Tang L, Xiong W, Ge H, Zhang H, Lu S, Shen Y, Wang J, Fei J, Wang Z (2019). Two novel testis-specific long noncoding RNAs produced by *1700121C10Rik* are dispensable for male fertility in mice.. J Reprod Dev.

[R14] Marques AC, Ponting CP (2014). Intergenic lncRNAs and the evolution of gene expression.. Curr Opin Genet Dev.

[R15] Melissari MT, Grote P (2016). Roles for long non-coding RNAs in physiology and disease.. Pflugers Arch.

[R16] Mollan, K.R., Trumble, I.M., Reifeis, S.A., Ferrer, O., Bay, C.P., Baldoni, P.L., and Hudgens, M.G. (2020). Precise and accurate power of the rank-sum test for a continuous outcome. Journal of Biopharmaceutical Statistics 30: 639-648. 10.1080/10543406.2020.1730866 PMC7316590 32126888

[R17] Rossi A, Kontarakis Z, Gerri C, Nolte H, Hölper S, Krüger M, Stainier DY (2015). Genetic compensation induced by deleterious mutations but not gene knockdowns.. Nature.

[R18] Shields EJ, Petracovici AF, Bonasio R (2019). lncRedibly versatile: biochemical and biological functions of long noncoding RNAs.. Biochem J.

[R19] Serobyan V, Kontarakis Z, El-Brolosy MA, Welker JM, Tolstenkov O, Saadeldein AM, Retzer N, Gottschalk A, Wehman AM, Stainier DY (2020). Transcriptional adaptation in *Caenorhabditis elegans*.. Elife.

[R20] Soumillon M, Necsulea A, Weier M, Brawand D, Zhang X, Gu H, Barthès P, Kokkinaki M, Nef S, Gnirke A, Dym M, de Massy B, Mikkelsen TS, Kaessmann H (2013). Cellular source and mechanisms of high transcriptome complexity in the mammalian testis.. Cell Rep.

[R21] Tzur YB, Winter E, Gao J, Hashimshony T, Yanai I, Colaiácovo MP (2018). Spatiotemporal Gene Expression Analysis of the *Caenorhabditis elegans* Germline Uncovers a Syncytial Expression Switch.. Genetics.

[R22] Ulitsky I, Bartel DP (2013). lincRNAs: genomics, evolution, and mechanisms.. Cell.

[R23] Washietl S, Kellis M, Garber M (2014). Evolutionary dynamics and tissue specificity of human long noncoding RNAs in six mammals.. Genome Res.

[R24] Wei S, Chen H, Dzakah EE, Yu B, Wang X, Fu T, Li J, Liu L, Fang S, Liu W, Shan G (2019). Systematic evaluation of C. elegans lincRNAs with CRISPR knockout mutants.. Genome Biol.

[R25] Wichman L, Somasundaram S, Breindel C, Valerio DM, McCarrey JR, Hodges CA, Khalil AM (2017). Dynamic expression of long noncoding RNAs reveals their potential roles in spermatogenesis and fertility.. Biol Reprod.

[R26] Zhang B, Arun G, Mao YS, Lazar Z, Hung G, Bhattacharjee G, Xiao X, Booth CJ, Wu J, Zhang C, Spector DL (2012). The lncRNA Malat1 is dispensable for mouse development but its transcription plays a cis-regulatory role in the adult.. Cell Rep.

[R27] Zhou Y, Zhang X, Xiong S, Zeng X, Zhang X (2021). Predicted gene 31453 (Gm31453) and the gene encoding carboxypeptidase A5 (Cpa5) are not essential for spermatogenesis and male fertility in the mouse.. Reprod Fertil Dev.

[R28] Zhu Y, Lin Y, He Y, Wang H, Chen S, Li Z, Song N, Sun F (2020). Deletion of lncRNA5512 has no effect on spermatogenesis and reproduction in mice.. Reprod Fertil Dev.

